# Attention Networks for the Quality Enhancement of Light Field Images

**DOI:** 10.3390/s21093246

**Published:** 2021-05-07

**Authors:** Ionut Schiopu, Adrian Munteanu

**Affiliations:** Department of Electronics and Informatics (ETRO), Vrije Universiteit Brussel (VUB), Pleinlaan 2, 1050 Brussels, Belgium; acmuntea@etrovub.be

**Keywords:** attention network, quality enhancement, light field images, video coding

## Abstract

In this paper, we propose a novel filtering method based on deep attention networks for the quality enhancement of light field (LF) images captured by plenoptic cameras and compressed using the High Efficiency Video Coding (HEVC) standard. The proposed architecture was built using efficient complex processing blocks and novel attention-based residual blocks. The network takes advantage of the macro-pixel (MP) structure, specific to LF images, and processes each reconstructed MP in the luminance (Y) channel. The input patch is represented as a tensor that collects, from an MP neighbourhood, four Epipolar Plane Images (EPIs) at four different angles. The experimental results on a common LF image database showed high improvements over HEVC in terms of the structural similarity index (SSIM), with an average Y-Bjøntegaard Delta (BD)-rate savings of 36.57%, and an average Y-BD-PSNR improvement of 2.301 dB. Increased performance was achieved when the HEVC built-in filtering methods were skipped. The visual results illustrate that the enhanced image contains sharper edges and more texture details. The ablation study provides two robust solutions to reduce the inference time by 44.6% and the network complexity by 74.7%. The results demonstrate the potential of attention networks for the quality enhancement of LF images encoded by HEVC.

## 1. Introduction

In recent years, the technological breakthroughs in the sensor domain have made possible the development of new camera systems with steadily increasing resolutions and affordable prices for users. In contrast to conventional Red-Green-Blue (RGB) cameras, which only capture light intensity, plenoptic cameras provide the unique ability of distinguishing between the light rays that hit the camera sensor from different directions using microlens technology. To this end, the main lens of plenoptic cameras focus light rays onto a microlens plane, and each microlens captures the incoming light rays from different angles and directs them onto the camera sensor.

For each microlens, a camera sensor produces a so-called Macro-Pixel (MP). The raw LF image contains the entire information captured by the camera sensor, where the array of microlenses generates a corresponding array of MPs, a structure also known as lenslet images. Since each pixel in the MP corresponds to a specific direction of the incoming light, the lenslet image is typically arranged as an array of SubAperture Images (SAIs), where each SAI collects, from all MPs, one pixel at a specific position corresponding to a specific direction of the incoming light. The captured LF image can, thus, be represented as an array of SAIs corresponding to a camera array with a narrow baseline.

LF cameras have proven to be efficient passive devices for depth estimation. A broad variety of depth estimation techniques based on LF cameras have been proposed in the literature, including multi-stereo techniques [1,2], artificial intelligence-based methods [3] as well as combinations of multi-stereo and artificial intelligence-based techniques [4]. Accurately estimating depth is of paramount importance in view synthesis [5] and 3D reconstruction [6,7].

The LF domain was intensively studied during recent decades, and many solutions were proposed for each module in the LF processing pipeline, such as LF acquisition, representation, rendering, display, and LF coding. The LF coding approaches are usually divided into two major classes, including transform-based approaches and predictive-based approaches, depending on which module in the image or video codec is responsible for exploiting the LF correlations.

The transform-based approaches are designed to apply a specific type of transform, such as Discrete Cosine Transform [8,9], Discrete Wavelet Transform [10,11], Karhunen Loéve Transform [12,13], or Graph Fourier Transform [14,15], to exploit the LF correlations.

However, the predictive-based approaches received more attention as they propose a more straightforward solution where different prediction methods are proposed to take advantage of the LF structure. These approaches propose to exploit the correlations between the SAIs using the coding tools in the High Efficiency Video Coding (HEVC) standard [16].

The pseudo-video-sequence-based approach proposes to select a set of evenly distributed SAIs as intra-coded frames and the remaining SAIs as inter-coded frames, e.g., [17,18]. In [19,20], the non-local spatial correlation is exploited when using the lenslet representation. The view-synthesis-based approach proposes to encode only a sparse set of reference SAIs and additional geometry information and then to synthesize the remaining SAIs at the decoder side [21,22]. In this work, we first employ HEVC [16] to encode the SAI video sequence and then to enhance the reconstructed lenslet image. The proposed Convolutional Neural Network (CNN)-based filtering method can be used to post-process any HEVC-based solution.

The attention mechanism was first proposed in the machine translation domain [23]. The main idea is that instead of building a single context vector, it is better to create weighted shortcuts between the context vector and the entire source input. This revolutionary concept now provides outstanding improvements in different domains, such as hyperspectral image classification [24], deblurring [25], image super-resolution [26], traffic sign recognition [27], and small object detection [28], to name a few. Many different network architectures have leveraged the attention mechanism to significantly improve over the state-of-the-art. In this work, an attention-based residual block is introduced to help the architecture learn and focus more on the most important information in the current MP context.

In our prior work, research efforts were invested to provide innovative solutions for LF coding based on efficient Deep-Learning (DL)-based prediction methods [20,29,30,31,32] and CNN-based filtering methods for quality enhancement [33,34]. In [29], we introduced a lossless codec for LF images based on context modeling of SAI images. In [30], we proposed an MP prediction method based on neural networks for the lossless compression of LF images.

In [31], we proposed to employ a DL-based method to synthesize an entire LF image based on different configurations of reference SAIs and then to employ an MP-wise prediction method to losslessly encode the remaining views. In [32], we proposed a residual-error prediction method based on deep learning and a context-tree based bit-plane codec, where the experimental evaluation was carried out on photographic images, LF images, and video sequences. In [20], the MP was used as an elementary coding unit instead of HEVC’s traditional block-based coding structure for lossy compression of LF images. In recent work, we focused on researching novel CNN-based filtering methods.

In [33], we proposed a frame-wise CNN-based filtering method for enhancing the quality of HEVC-decoded videos. In [34], we proposed an MP-wise CNN-based filtering method for the quality enhancement of LF images. The goal of this paper is to further advance our findings in [34] by introducing a novel filtering method based on attention networks, where the proposed architecture is built based on efficient processing blocks and attention-based residual blocks and operates on Epipolar Plane Images (EPI)-based input patches.

In summary, the novel contributions of this paper are as follows:

(1) A novel CNN-based filtering method is proposed for enhancing the quality of LF images encoded using HEVC [16].
(2) A novel neural network architecture design for the quality enhancement of LF images is proposed using an efficient complex Processing Block (PB) and a novel Attention-based Residual Block (ARB).
(3) The proposed CNN-based filtering method follows an MP-wise filtering approach to take advantage of the specific LF structure.
(4) The input patch is designed as a tensor of four MP volumes corresponding to four EPIs at four different angles ($0^{\circ },45^{\circ },90^{\circ },$ and $135^{\circ }$).
(5) The elaborated experimental validation carried out on the EPFL LF dataset [35] demonstrates the potential of attention networks for the quality enhancement of LF images.

The remainder of this paper is organized as follows. Section 2 presents an overview of the state-of-the-art methods for quality enhancement. In Section 3, we describe the proposed CNN-based filtering method. Section 4 presents the experimental validation on LF images. Finally, in Section 5, we draw our conclusions from this work.

## 2. Related Work

In recent years, many coding solutions based on machine learning techniques have rapidly gained popularity by proposing to simply replace specific task-oriented coding tools in the HEVC coding framework [16] with powerful DL-based equivalents. The filtering task was widely studied, and many DL-based filtering tools for quality enhancement were introduced to reduce the effects of coding artifacts in the reconstructed video.

The first DL-based quality enhancement tools were proposed for image post-filtering. In [36], the Artifact Reduction CNN (AR-CNN) architecture was proposed to reduce the effect of the coding artifacts in JPEG compressed images. In [37], a more complex architecture with hierarchical skip connections was proposed. A dual (pixel and transform) domain-based filtering method was proposed in [38]. A discriminator loss, as in Generative Adversarial Networks (GANs), was proposed in [39]. An iterative post-filtering method based on a recurrent neural network was proposed in [40].

Inspired by AR-CNN [36], the Variable-filter-size Residue-learning CNN (VRCNN) architecture was proposed in [41]. The inter-picture correlation is used by processing multiple neighboring frames to enhance one frame using a CNN [42]. In [43], the authors proposed to make use of mean- and boundary-based masks generated by HEVC partitioning. In [44], a CNN processes the intra prediction signal and the decoded residual signal. In [45], a CNN processes the QP value and the decoded frame. In [46], the CNN operates on input patches designed based on additional information extracted from the HEVC decoder, which specifies the current QP value and the CU partitioning maps.

In another approach, the authors proposed to replace the HEVC built-in in-loop filtering, the Deblocking Filter (DBF) [47], and the Sample Adaptive Offset (SAO) [48]. This is a more demanding task as, in this case, the filtered frame enters the coding loop and serves as a reference to other frames. In [49], a CNN was used to replace the SAO filter. Similarly, in [50], a deep CNN was applied after SAO and was controlled by the frame- and coding tree unit (CTU)-level flags.

In [51], the authors used a deep residual network to estimate the lost details. In [52], the Multistage Attention CNN (MACNN) architecture was introduced to replace the HEVC in-loop filters. Other coding solutions focus on inserting new filtering blocks in the HEVC framework. In [53], an adaptive, in-loop filtering algorithm was proposed using an image nonlocal prior, which collaborates with the existing DBF and SAO in HEVC. In [54], a residual highway CNN (RHCNN) was applied after the SAO filter. In [55], a content-aware CNN-based in-loop filtering method was integrated in HEVC after the SAO built-in filter.

In this work, we propose to employ the attention mechanism for the quality enhancement of LF images (represented as lenslet images) by following an MP-wise filtering approach. Our experiments show that an increased coding performance was achieved when the SAI video sequence was encoded by running HEVC without its built-in filtering methods, DBF [47] and SAO [48].

## 3. Proposed Method

In the literature, the LF image is usually represented as a 5D structure denoted by LF(p,q,x,y,c), where the (p,q) pair denotes the pixel location in an MP matrix, usually of N×N resolution; the (x,y) pair denotes the pixel location in an SAI matrix of size W×H; and *c* denotes the primary color channel, c=1,2,3. Let us denote $MP_{x,y}=LF\left(:,:,x,y,c\right)$ as the MP captured by the microlens at position (x,y) in the microlens array; $SAI_{p,q}=LF\left(p,q,:,:,c\right)$ as the SAI corresponding to view (p,q) in the SAI stack; and LL as the lenslet image of size NH×NW, which is defined as follows:

(1) $$LL\left(\left(x-1\right)N+1:xN,\left(y-1\right)N+1:yN,c\right)=MP_{x,y},\forall x=1:W,\forall y=1:H.$$

The experiments were conducted using the EPFL LF dataset [35] where N=15 and W×H=625×434. The LF images were first color-transformed from the RGB color-space to the YUV color-space, and only the Y (luminance) channel was enhanced. Therefore, c=1 and $MP_{x,y}$ were of size 15×15.

**Figure 1:**
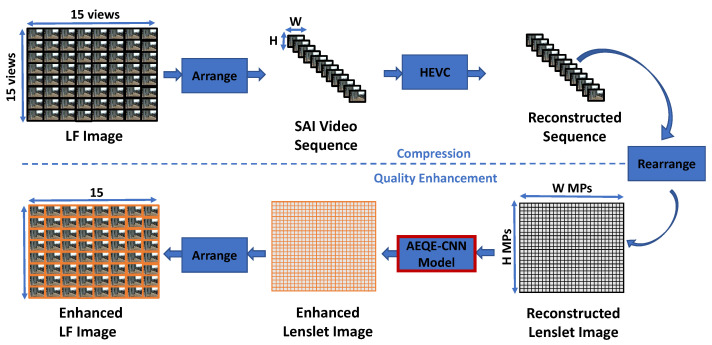
The proposed CNN-based filtering scheme. (**Top**) Compression: The LF Image (represented as an array of SAI) is arranged as a SAI video sequence and then encoded by HEVC. (**Bottom**) Quality Enhancement: The reconstructed sequence is arranged as a lenslet image (represented as an array of MPs) and each MP is enhanced by the proposed CNN-based filtering method using an AEQE-CNN model.

In this paper, a novel CNN-based filtering method is proposed to enhance the quality of LF images encoded using the HEVC video coding standard [16]. Figure 1 depicts the proposed CNN-based filtering scheme. The LF image, represented as an array of SAIs, is first arranged as an SAI video sequence and then encoded by the reference software implementation of HEVC called HM (HEVC Test Model) [56] under the All Intra (AI) profile [57]. Any profile can be used to encode the SAI video sequence as the proposed CNN-based filtering scheme is applied to the entire SAI video sequence. Therefore, in this work, a raster scan order is used to generate the SAI video sequence, while in the literature, a spiral order starting from the center view and looping in a clockwise manner towards the edge views is used to generate the SAI video sequence. Next, the reconstructed SAI sequence is arranged as a lenslet image using Equation (1), and EPI-based input patches were extracted from the reconstructed lenslet image, see Section 3.1.

A CNN model with the proposed novel deep neural architecture called Attention-aware EPI-based Quality Enhancement Convolutional Neural Network (AEQE-CNN), see Section 3.2, processed the input patches to enhance the MPs and obtain the enhanced lenslet image. Finally, the enhanced lenslet image is arranged as a LF image to be easily consumed by users.

Section 3.1 presents the proposed algorithm used to extract the EPI-based input patches. Section 3.2 describes in detail the network design of the proposed AEQE-CNN architecture. Section 3.3 presents the training details.

### 3.1. Input Patch

**Figure 2:**
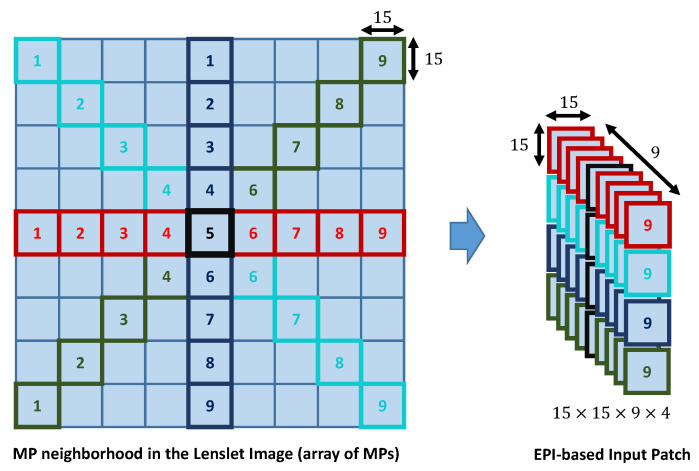
Extraction of the EPI-based input patch from the lenslet image represented as an array of MPs. Four EPIs are selected: $0^{\circ }$ (horizontal) EPI marked with red, $45^{\circ }$ (first diagonal) EPI marked with cyan, $90^{\circ }$ (vertical) EPI marked with blue, and $135^{\circ }$ (second diagonal) EPI marked with green. The current MP is marked with black.

In this paper, input patches of size 15×15×9×4 were extracted from the reconstructed lenslet image. More exactly, the input patch concatenated four EPIs corresponding to $0^{\circ }$ (horizontal EPI), $45^{\circ }$ (first diagonal EPI), $90^{\circ }$ (vertical EPI), and $135^{\circ }$ (second diagonal EPI) from the MP neighbourhood of b=4 MPs around the current MP, as depicted in Figure 2. Let us denote $N_{x,y}$ as the MP neighbourhood around the current MP, $MP_{x,y},$ where

(2) $$N_{x,y}=\left[\begin{array}{ccccc} MP_{x-b,y-b} & \ldots & MP_{x-b,y} & \ldots & MP_{x-b,y+b}\\ \vdots & \; & \vdots & \; & \vdots \\ MP_{x,y-b} & \ldots & MP_{x,y} & \ldots & MP_{x,y+b}\\ \vdots & \; & \vdots & \; & \vdots \\ MP_{x+b,y-b} & \ldots & MP_{x+b,y} & \ldots & MP_{x+b,y+b} \end{array}\right].$$

Four EPIs of size N×N×(2b+1)=15×15×9 were extracted from $N_{x,y}$ as follows:

(1) The $0^{\circ }$ EPI of MP volume: $[MP_{x,y-b}\;MP_{x,y-b+1}\;\ldots \;MP_{x,y+b}];$
(2) The $45^{\circ }$ EPI of MP volume: $[MP_{x-b,y-b}\;MP_{x-b+1,y-b+1}\;\ldots \;MP_{x+b,y+b}];$
(3) The $90^{\circ }$ EPI of MP volume: $[MP_{x-b,y}\;MP_{x-b+1,y}\;\ldots \;MP_{x+b,y}];$ and
(4) The $135^{\circ }$ EPI of MP volume: $[MP_{x+b,y-b}\;MP_{x+(b-1),y-(b-1)}\;\ldots \;MP_{x-b,y+b}].$

The four EPIs were processed separately by the AEQE-CNN architecture as described in the following section.

### 3.2. Network Design

**Figure 3:**
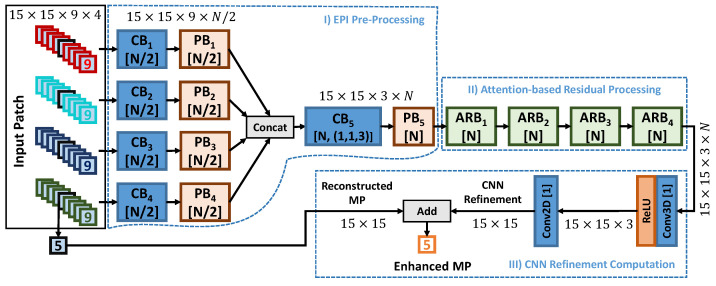
The proposed network architecture called Attention-aware EPI-based Quality Enhancement Convolutional Neural Network (AEQE-CNN).

Figure 3 depicts the proposed deep neural network architecture. AEQE-CNN is designed to process the EPI-based input patches using efficient processing blocks and attention-based residual blocks. 3D Convolutional layers (Conv3D) equipped with 3×3×3 kernels are used throughout the network architecture.

**Figure 4:**
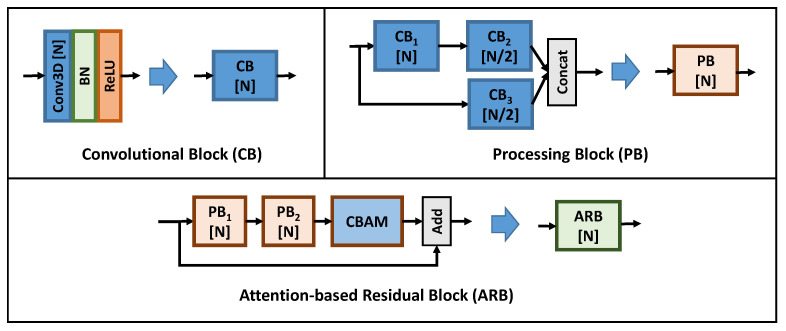
The layer structure of the three blocks used to build the proposed architecture: (**top-left**) Convolutional Block (CB); (**top-right**) Processing Block (PB); and (**bottom**) Attention-based Residual Block (APB), where the Convolutional Block Attention Module (CBAM) was proposed [59] and modified here as depicted in Figure 5.

**Figure 5:**
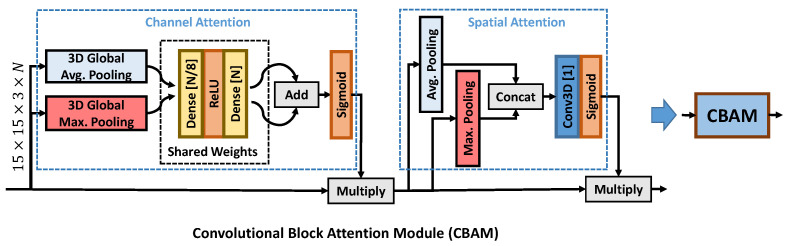
The layer structure of Convolutional Block Attention Module (CBAM), which uses both channel and spatial attention. The module was proposed in [59] and was modified here to compute the attention map for an MP volume.

AEQE-CNN was built using the following types of blocks depicted in Figure 4: *(i)* the Convolutional Block (CB) contains a sequence of one Conv3D, one batch normalization (BN) layer [58], and one Rectified Linear Unit (ReLU) activation function; *(ii)* the proposed Processing Block (PB) contains a two branch design with one and two CB blocks where the feature maps of the two branches are concatenated to obtain the output feature maps; *(iii)* the proposed Attention-based Residual Block (APB) contains a sequence of two PB blocks and one Convolutional Block Attention Module (CBAM), see Figure 5, and a skip connection to process the current patch.

Figure 3 shows that the AEQE-CNN architecture processes the EPI-based input patch using three stages. In the first stage, called EPI Pre-Processing, the MP volume corresponding to an EPI is processed using one CB block and one PB block, each equipped with N/2 filters, to extract the EPI feature maps, which are then concatenated and further processed by $CB_{5}$ and $PB_{5},$ which are both equipped with *N* filters. $CB_{5}$ uses the stride s=(1,1,3) to reduce the current patch resolution from 15×15×9 to 15×15×3 to decrease the inference time and to reduce the MP neighbourhood from 9 MPs to 3 MPs.

In the second stage, called Attention-based Residual Processing, a sequence of four APB blocks with *N* filters are used to further process the patch and extract the final feature maps of size 15×15×N. The final stage, called CNN Refinement Computation, is used to extract the final CNN-refinement using one Conv3D layer with ReLU activation and one Conv2D layer (equipped with a 3×3 kernel) with one filter. The CNN-refinement is then added to the currently reconstructed MP to obtain the enhanced MP.

In this paper, we propose to employ an attention-based module designed based on the CBAM module introduced in [59]. Figure 5 depicts the layer structure of CBAM. CBAM proposes the use of both channel attention and spatial attention. The channel attention uses the shared weights of two dense layers to process the two feature vectors extracted using global average pooling and global maximum pooling, respectively. The spatial attention uses a Conv3D layer to process the feature maps extracted using average pooling and maximum pooling. The two types of attention maps are obtained using a sigmoid activation layer and then applied in turn using a multiplication layer. The CBAM block was proposed in [59] for the processing of two-dimensional patches, while, here, the CBAM design was modified to be applied to MP volumes (three-dimensional patches).

### 3.3. Training Details

The AEQE-CNN models were trained using the Mean Squared Error (MSE) loss function equipped with an $\ell_{2}$ regularization procedure to prevent model over-fitting. Let us denote: $\Theta_{\mathrm{AEQE}-\mathrm{CNN}}$ as the set of all learned parameters of the AEQE-CNN model; $X^{\left(i\right)}$ as the *i*-th EPI-based input patch in the training set of size 15×15×9×4; and $Y^{\left(i\right)}$ as the corresponding MP in the original LF image of size 15×15. Let F(·) be the function that processes $X^{\left(i\right)}$ using $\Theta_{\mathrm{AEQE}-\mathrm{CNN}}$ to compute the enhanced MP as ${\hat{Y}}^{\left(i\right)}=F\left(X^{\left(i\right)},\Theta_{\mathrm{AEQE}-\mathrm{CNN}}\right).$ The loss function is formulated as follows:

(3) $$L\left(\Theta_{\mathrm{AEQE}-\mathrm{CNN}}\right)=\frac{1}{L}\sum_{i=1}^{L}∥vec\left(Y^{\left(i\right)}\right)-vec\left({\hat{Y}}^{\left(i\right)}\right){∥}_{2}^{2}+\lambda ||\Theta_{\mathrm{AEQE}-\mathrm{CNN}}{\left|\right|}_{2}^{2},$$

where *L* is the number of input patches, λ is the regularization term that is set empirically as λ=0.001, and vec is the vectorization operator. Here, the Adam optimization algorithm [60] is employed.

By setting N=32, the AEQE-CNN models contain 782,661 parameters that must be trained. Experiments using a more lightweight AEQE-CNN architecture were also performed, see Section 4.4. Version *HM 16.18* of the reference software implementation is used for the HEVC codec [16]. Note that other software implementations of HEVC, such as FFmpeg [61], Kvazaar [62], and OpenHEVC [63,64] are available; however, in this work, the reference software implementation of HEVC was used due to its high popularity within the research community. The proposed CNN-based filtering method trained four AEQE-CNN models, one for each of the four standard QP values, QP={22,27,32,37}.

The proposed neural network was implemented in the Python programming language using the Keras open-source deep-learning library, and was run on a machine equipped with Titan Xp Graphical Processing Units (GPUs).

In our previous work [33,34], the experimental results showed that an improved performance was obtained when HEVC was modified to skip its built-in in-loop filters, DBF [47] and SAO [48]. Therefore, here, four models were trained using EPI-based input patches extracted from reconstructed LF images obtained by running HEVC with its built-in in-loop filters, called AEQE-CNN + DBF&SAO, and four models were trained using EPI-based input patches extracted from reconstructed LF images obtained by running HEVC without its built-in in-loop filters, called AEQE-CNN. This training strategy demonstrates that the proposed CNN-based filtering method can be integrated into video coding systems where no modifications to the HEVC anchor are allowed.

The proposed AEQE-CNN architecture differs from our previous architecture design named MP-wise quality enhancement CNN (MPQE-CNN) [34] as follows. MPQE-CNN operates on MP volumes extracted from the closest 3×3 MP neighbourhood, while AEQE-CNN operates on EPI-based input patches extracted from an 9×9 MP neighbourhood. MPQE-CNN follows a multi-resolution design with simple CB blocks, while AEQE-CNN follows a design of multi-EPI branch processing and sequential residual block processing built based on more efficient PB blocks and novel attention-aware ARB blocks.

## 4. Experimental Validation

Section 4.1 describes the experimental setup used to compare the proposed CNN-based filtering method with the state-of-the-art methods. Section 4.2 illustrates the experimental results obtained over the test. Section 4.3 presents the visual results of the proposed CNN-based filtering method in comparison with the HEVC anchor. Finally, Section 4.4 presents an ablation study that analyses the possibility to reduce the network complexity and runtime using different approaches.

### 4.1. Experimental Setup

**LF image Dataset.** The experimental validation was carried out on the EPFL LF dataset [35], which contained 118 LF images in the RGB format, divided into 10 categories. Similar to [34], here, only the first 8 bits of the RGB color channels were encoded, and, similar to [29], 32 corner SAIs (8 from each corner) were dropped from the array of SAIs as they contained sparse information due to the shape of the microlens used by the plenoptic camera. Since the SAIs were color-transformed to the YUV format and only the Y channel was enhanced, the SAI video sequence contained 193 Y-frames. The closest frame resolution that HEVC [16] accepted as input was W×H=632×440.

For a fair comparison with MPQE-CNN [34], the experiments were carried out on the same Training set (10 LF images) and Test set (108 LF images) as defined in [34], i.e., the Training set contained the following LF images: *Black_Fence*, *Chain_link_fence_1*, *ISO_chart_1*, *Houses_&_lake*, *Backlight_1*, *Broken_mirror*, *Bush*, *Fountain_&_Vincent_1*, *Ankylosaurus_&_Diplodocus_1*, and *Bench_in_Paris*. A total number of 625×434×10=2,712,500 EPI-based input patches were collected from the 10 training images, and a 90%−10% ratio was used for splitting the training set into training−validation data. A batch size of 350 EPI-based input patches was used.

**Comparison with the state-of-the-art methods.** The two proposed methods, AEQE-CNN + DBF&SAO and AEQE-CNN, were compared with *(i)* the HEVC [16] anchor, denoted by HEVC + DBF&SAO; *(ii)* the FQE-CNN architecture from [33] where each SAI in the LF image was enhanced in turn; and *(iii)* the MPQE-CNN architecture from [34] based on a similar MP-wise filtering approach. The distortion was measured using the Peak Signal-to-Noise Ratio (PSNR) and the Structural Similarity Index Measure (SSIM) [65]. The standard Bjøntegaard delta bitrate (BD-rate) savings and Bjøntegaard delta PSNR (BD-PSNR) improvement [66] were computed using the four standard QP values: QP={22,27,32,37}.

### 4.2. Experimental Results

**Figure 6:**
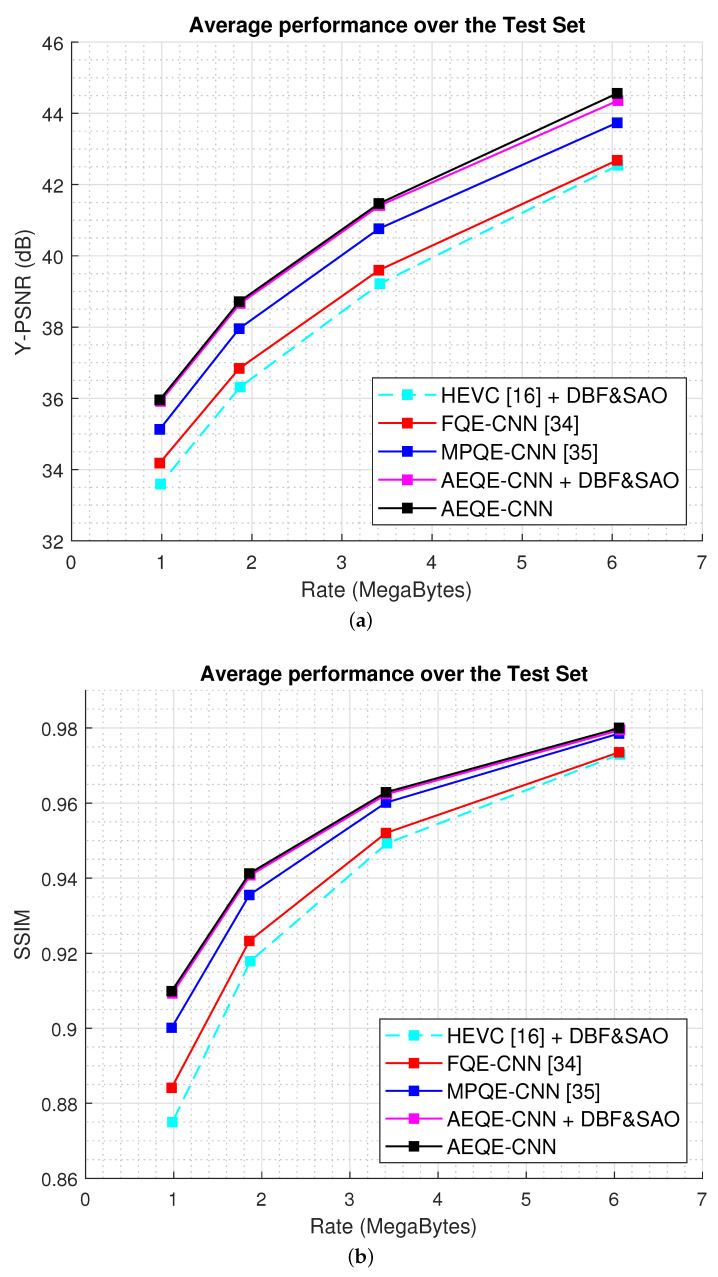
The Rate-Distortion results over the test set. (**a**) Y-PSNR-vs.-bitrate. (**b**) SSIM-vs.-bitrate.

**Figure 7:**
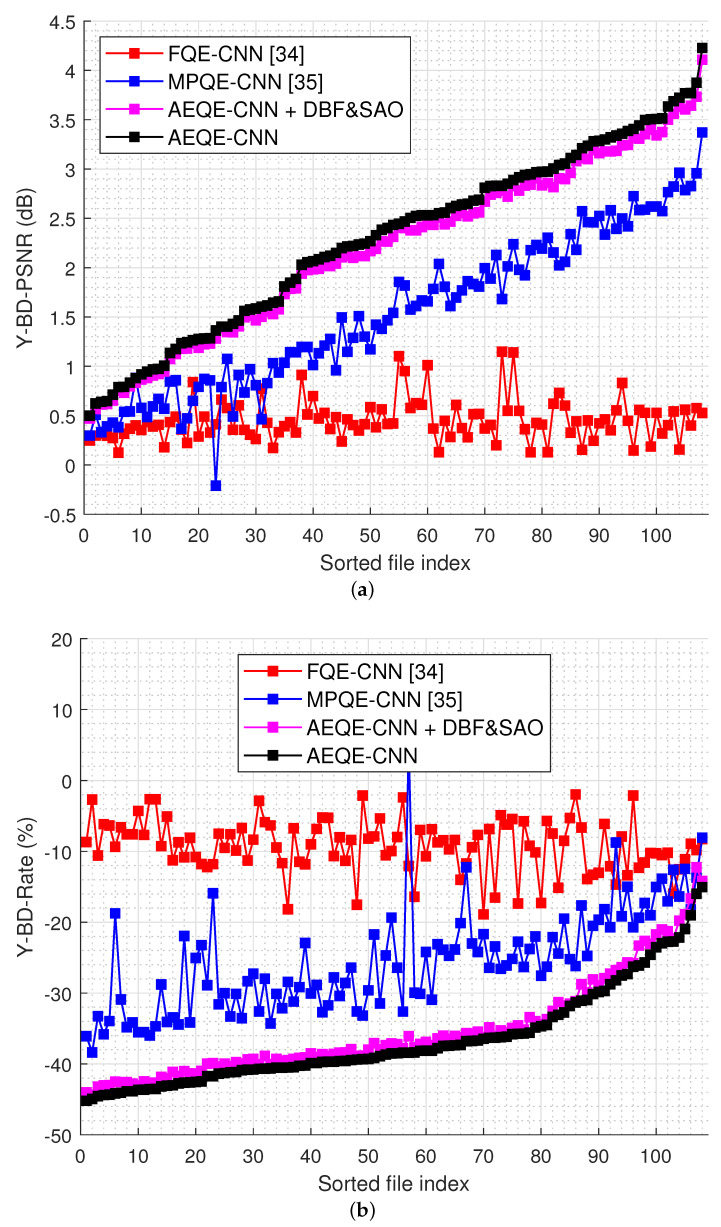
The Bjøntegaard metric results for every LF image in the test set: (**a**) Y-BD-PSNR gains (dB); (**b**) Y-BD-rate savings (%).

Figure 6 shows the compression results over the test set (108 LF images) for the rate-distortion curves computed as Y-PSNR-vs.-bitrate and SSIM-vs.-bitrate. Figure 7 shows the Y-BD-PSNR and Y-BD-rate values computed for each LF image in the test set. The proposed methods provide an improved performance compared with HEVC [16] + DBF&SAO, FQE-CNN [33], and MPQE-CNN [34] at both low and high bitrates. The results show that AEQE-CNN provided a small improvement over AEQE-CNN + DBF&SAO. The proposed CNN-based filtering method was able to provide a large improvement even when no modification was applied to the HEVC video codec.

Table 1 shows the average results obtained over the test set. AEQE-CNN provided Y-BD-rate savings of 36.57% and Y-BD-PSNR improvements of 2.301 dB over HEVC [16], i.e., a more than 40% improvement was achieved compared with MPQE-CNN [33].

**Figure 8:**
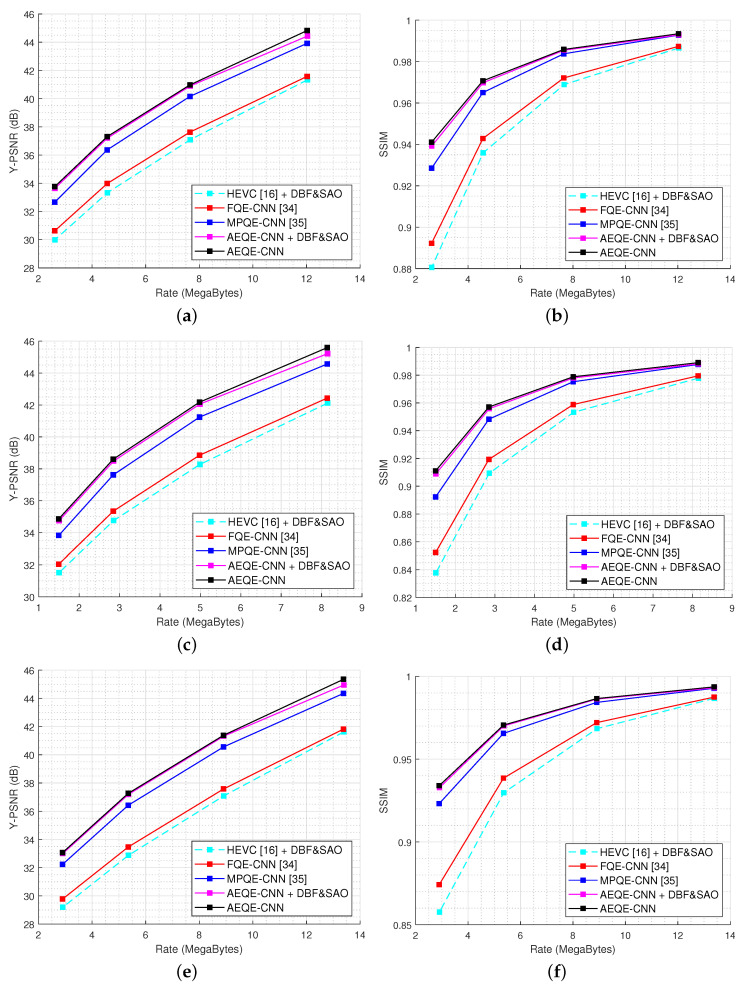
The Rate-Distortion results for three LF images in the test set. (**a**) Y-PSNR-vs.-bitrate for *Chain_link_fence_2*; (**b**) SSIM-vs.-bitrate for *Chain_link_fence_2*; (**c**) Y-PSNR-vs.-bitrate for *Flowers*; (**d**) SSIM-vs.-bitrate for *Flowers*; (**e**) Y-PSNR-vs.-bitrate for *Palais_du_Luxembourg*; (**f**) SSIM-vs.-bitrate for *Palais_du_Luxembourg*.

Figure 8 shows the Rate-Distortion (RD) results for three randomly selected LF images in the test set, *Chain_link_fence_2*, *Flowers*, and *Palais_du_Luxembourg*. AEQE-CNN provided an Y-BD-PSNR improvement of around 2 dB at both low and high bitrates. The SSIM-vs.-bitrate results show that the visual quality at low bitrates was highly improved of around 0.08.

### 4.3. Visual Results

**Figure 9:**
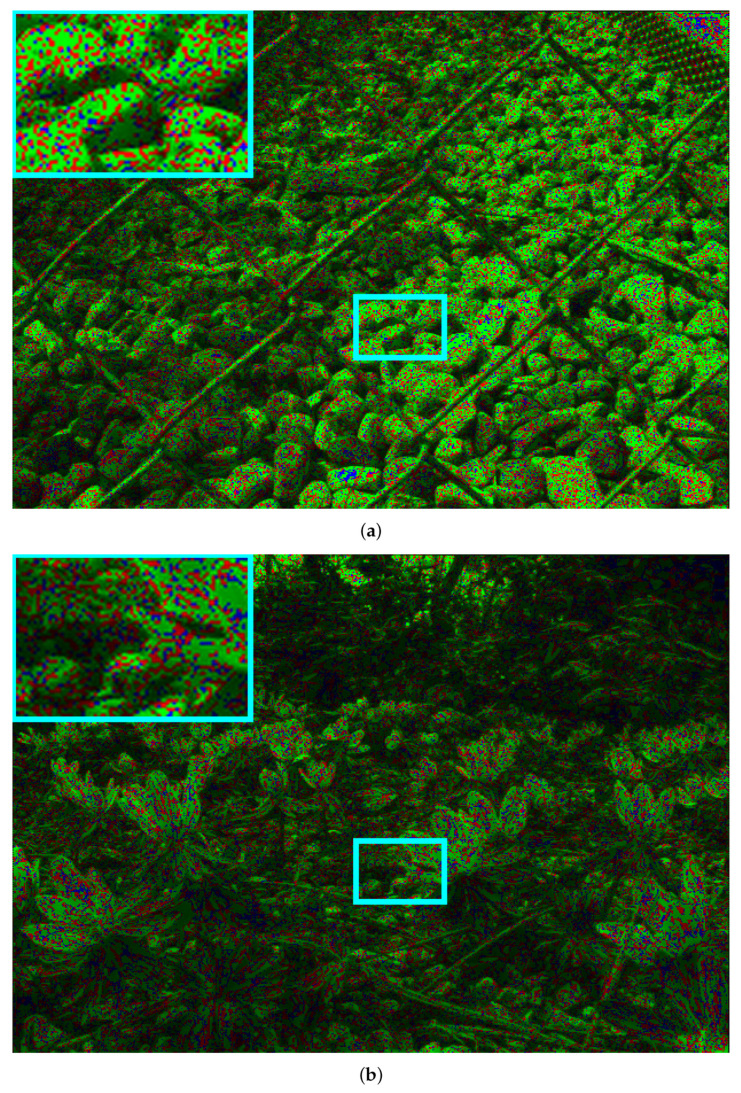
Pseudo-coloured image comparison between AEQE-CNN and HEVC [16] + DBF&SAO based on the absolute reconstruction error for the center SAI at position (p,q)=(8,8), and for QP=37. Green marks the pixel positions where AEQE-CNN achieved better performance. Blue marks the pixel positions where the two methods had the same performance. Red marks pixels where HEVC [16] + DBF&SAO achieved better performance. The cyan rectangle marks an image area shown zoomed-in at the top-left corner and the corresponding Y channel in Figure 10. The results for two LF images in the test set: (**a**) *Chain_link_fence_2*; (**b**) *Flowers*.

Figure 9 shows the pseudo-coloured image comparison between AEQE-CNN and HEVC [16] + DBF&SAO for two LF images in the test set, *Chain_link_fence_2* and *Flowers*. The green, blue, and red pixels mark the positions where AEQE-CNN provided an improved, similar, and worse performance, respectively, compared with HEVC [16] + DBF&SAO anchor. Green is the dominant color, which shows that AEQE-CNN enhanced the quality of almost all pixels in the LF image.

**Figure 10:**
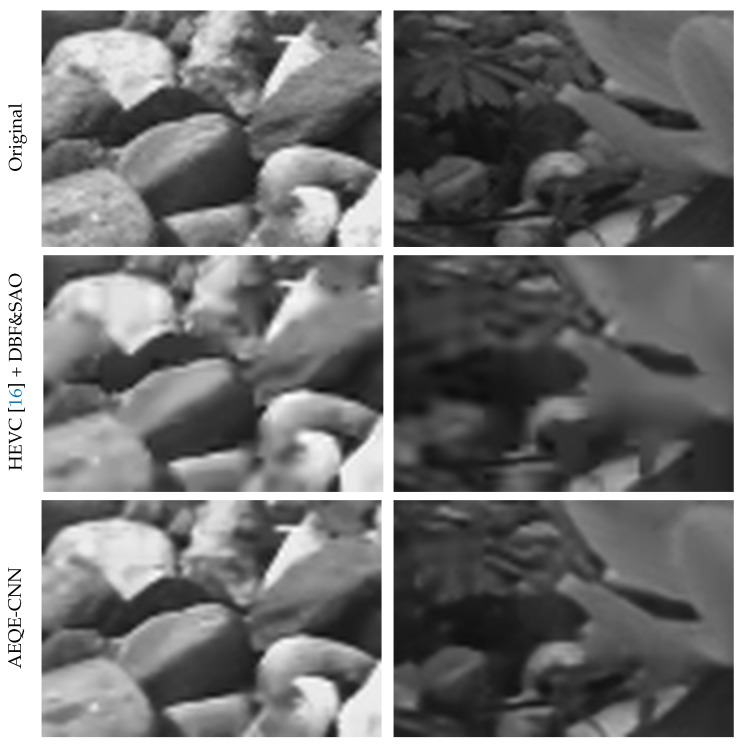
Visual comparison between AEQE-CNN and HEVC [16] + DBF&SAO for the Y channel of the zoomed-in image area marked by the cyan rectangle in Figure 9 above.

Figure 10 shows the visual result comparison between AEQE-CNN and HEVC [16] + DBF&SAO for the corresponding Y channel of the two zoomed-in image areas marked by cyan rectangles in Figure 9. AEQE-CNN provided much sharper image edges and added more details to the image textures.

### 4.4. Ablation Study

In this work, we also studied the possibility to reduce the network complexity and runtime using two different approaches. In the first approach, an architecture variation of AEQE-CNN was generated by halving the number of channels used throughout the architecture by the 3D Convolution layers from N=32 to N=16. This first AEQE-CNN architecture variation is called AEQE-CNN [N=16]. In the second approach, the size of the MP neighbourhood, $N_{x,y}$ (see Section 3.1), was reduced from 9×9 MPs (i.e., b=4) to 3×3 MPs (i.e., b=1).

More precisely, the same neighbourhood window as in [34] was used here with the goal of evaluating the influence of the size of the MP neighbourhood in the final enhancement results. In this case, the EPI volumes were of the size 15×15×3; therefore, the $CB_{5}$ block in the AEQE-CNN architecture (see Figure 3) used a default stride of $s^{\prime }=\left(1,\;1,\;1\right)$ instead of s=(1,1,3). This second AEQE-CNN architecture variation is called AEQE-CNN [3×3].

Table 2 shows the average results obtained over the test set for the three AEQE-CNN architectures. The AEQE-CNN provided the best performance using the highest complexity and runtime. The network variations corresponding to the two approaches for complexity reduction still provided a better performance compared with the state-of-the-art methods and a close performance to AEQE-CNN. AEQE-CNN [N=16] offered a reduction of 44.6% in the inference runtime and a reduction of 74.7% in the network complexity, with a drop in the average performance of only 8.93% in Y-BD-PSNR and 3.59% in Y-BD-Rate.

AEQE-CNN [3×3] offered a reduction of 40.7% in the inference runtime, with a drop in the average performance of only 9.6% in Y-BD-PSNR and of 4.05% in Y-BD-Rate. The ablation study demonstrate that AEQE-CNN [3×3] provided a large reduction in the network complexity and inference runtime while accepting a small performance drop compared with AEQE-CNN.

**Figure 11:**
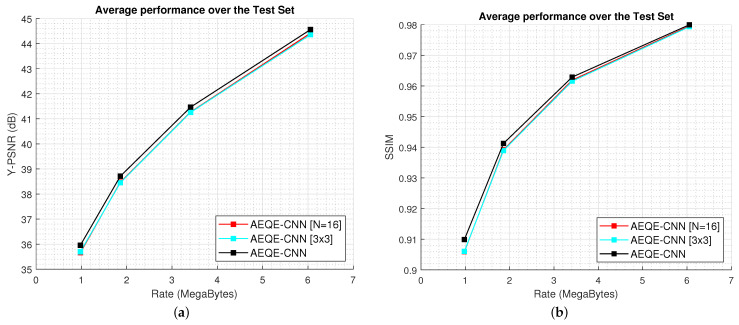
The Rate-Distortion results over the test set for the three network variations. (**a**) Y-PSNR-vs.-bitrate. (**b**) SSIM-vs.-bitrate.

Figure 11 shows the rate-distortion curves computed over the test set for AEQE-CNN [N=16], AEQE-CNN [3×3], and AEQE-CNN. The results demonstrate again that the two network variations provided a close performance to AEQE-CNN. The performance dropped with less than 0.2 dB at low and high bitrates for the two architecture variations. The results obtained by AEQE-CNN [3×3] demonstrate that the proposed AEQE-CNN architecture, built using the PB and ARB blocks, provided an improved performance compared with the MPQE-CNN architecture [34] when operating on the same MP neighbourhood.

**Figure 12:**
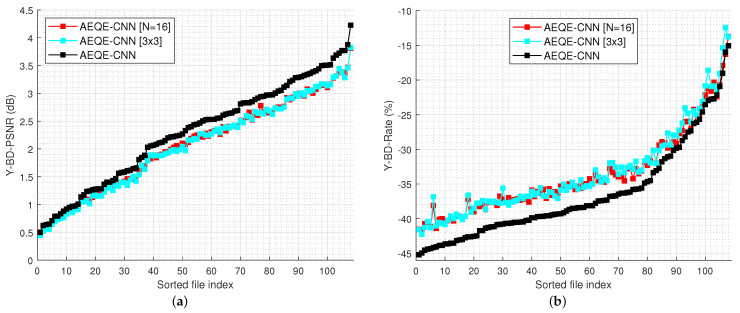
Bjøntegaard metrics results for every LF image in test set for the three network variations: (**a**) Y-BD-PSNR gains; (**b**) Y-BD-Rate savings.

Figure 12 shows the results of the Bjøntegaard metrics, Y-BD-PSNR and Y-BD-rate, computed for each LF image in the test set. The results demonstrate again that the two network variations provided a close performance to AEQE-CNN.

## 5. Conclusions

In this paper, we proposed a novel CNN-based filtering method for the quality enhancement of LF images compressed by HEVC. The proposed architecture, AEQE-CNN, was built using novel layer structure blocks, such as complex processing blocks and attention-based residual blocks. AEQE-CNN operated on an EPI-based input patch extracted from an MP neighbourhood of 9×9 MPs and followed an MP-wise filtering approach that was specific to LF images. Similar to previous research works, the proposed AEQE-CNN filtering method provided an increased performance when the conventional HEVC built-in filtering methods were skipped. The results demonstrate the high potential of attention networks for the quality enhancement of LF images.

In our future work, we plan to study different strategies to reduce the inference runtime using lightweight neural network architectures, and to employ the CNN-based filtering method to enhance the quality of the light field images compressed using other video codecs, such as AV1 and VVC.

## Figures and Tables

**Table 1:** Average results obtained over the test set.

Method	Bjøntegaard Metric	
	Y-BD-PSNR (dB)	Y-BD-Rate (%)
FQE-CNN [33]	0.4515	−9.1921
MPQE-CNN [34]	1.5478	−25.5285
AEQE-CNN + DBF&SAO	2.2044	−35.3142
AEQE-CNN	**2.3006**	**−36.5713**

**Table 2:** The average results obtained over the test set for the three AEQE-CNN network variations.

Method	Bjøntegaard Metric		Nr. of Trained	Inference Time
	Y-BD-PSNR	Y-BD-Rate	Parameters	Per Img.
AEQE-CNN [N=16]	2.0954 dB	–35.2581%	**197,661** (−74.7%)	**98 s** (−44.6%)
AEQE-CNN [3×3]	2.0799 dB	–35.0914%	782,661	105 s (−40.7%)
AEQE-CNN	**2.3006 dB**	**−36.5713%**	782,661	177 s
